# Occupational Health: An Ugly Picture for Flower Workers and Their Children

**DOI:** 10.1289/ehp.114-a463a

**Published:** 2006-08

**Authors:** David A. Taylor

Every year, Americans spend nearly $20 billion on fresh flowers, about 70% of which come from abroad, mainly from Latin America, according to the Society of American Florists. While this can represent an economic boon for some countries, overuse of pesticides and lack of protections for female workers can cause serious health effects for those women’s children, according to a paper in the March 2006 issue of *Pediatrics*.

The study of female workers in Ecuador’s flower industry and their children found that a mother’s work exposure to pesticides during pregnancy was associated with neurological impairment, including a significant decrease in visuospatial performance. After accounting for other factors such as malnutrition, the researchers concluded that “prenatal pesticide exposure may adversely affect brain development.”

The authors, led by Philippe Grandjean, an adjunct professor at the Harvard School of Public Health, also found that children whose mothers were exposed during pregnancy tended to have higher blood pressure than unexposed children, a finding with broader implications. “Increased blood pressure, when present in childhood, is a risk factor for cardiovascular disease in later life,” the researchers noted.

The researchers looked at schoolchildren under the age of 10 in the Andean community of Tabacundo. Physical exams checked each child’s blood pressure and certain neurobehavioral functions, such as motor coordination, dexterity, attention, short-term memory, balance, and spatial perception and performance. Mothers were interviewed about their own exposure history and background as well as their children’s medical history and health. The data analysis took into account each family’s housing and nutritional situation, as well as maternal education. The researchers also measured current pesticide exposure among the children.

Of 72 children included in the analysis, 37 were considered to have been exposed prenatally—they were born to women who had worked in the floriculture industry while pregnant. All of these mothers reported following normal safety precautions, and none had worked as pesticide applicators. Nineteen of the exposed children’s fathers and 16 of the unexposed children’s fathers also had worked in floriculture during the pregnancy, while most other fathers worked in construction trades.

Prenatal exposure was associated with significantly higher systolic blood pressure and substantial deficits on spatial performance. In this regard, the researchers concluded that pesticide toxicity may add to the adverse influence of malnutrition. Also, the effects of prenatal pesticide exposure seemed to last longer than those known to be associated with pesticide exposures in adults. However, the investigators found no link between prenatal exposure and stunting.

Elizabeth Guillette, an anthropologist at the University of Florida who has studied the health effects of pesticides in Mexico, says Grandjean’s study reinforces earlier findings. “Pesticide use is definitely impacting the offspring in terms of mental and neurophysical abilities,” she says.

Such concerns motivated the founders of Organic Bouquet, which since January 2001 has marketed flowers produced with fewer toxic pesticides. It sells flowers online and in natural food stores such as Whole Foods, using only producers certified by one of three programs. VeriFlora, one of the three certification programs, sets criteria for U.S.-sold flowers that include low pesticide residue and compliance with local labor laws.

As for traditional flower farms, Guillette says much better education is needed—not just on safe use at work, but also safe practices in the home, such as washing exposed clothes separately and minimizing in-home pesticide use. Grandjean agrees that education would help, but only if industry and individuals follow through with less extensive fumigations at work, use of less-toxic chemicals at work and at home, and use of protective equipment.

“I’m optimistic we can do something and change,” says Guillette, “but action needs to be taken now.”

## Figures and Tables

**Figure:**
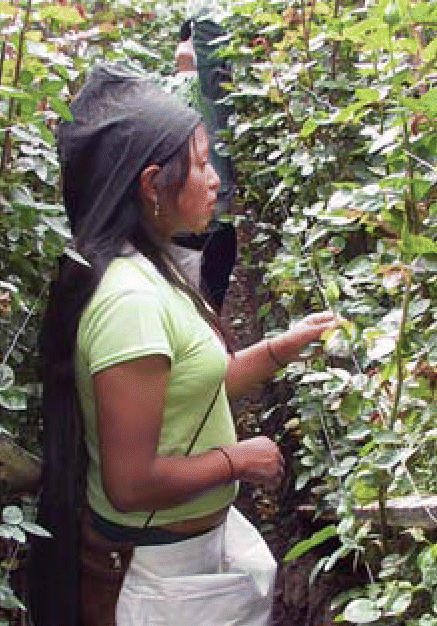
Nipping hazards in the bud Use of protective equipment while pregnant can curb ill effects in the children of floriculture workers.

